# Differences in Cancer Mortality Trends between Metropolitan and Non-Metropolitan Areas in Japan, 1999–2018

**DOI:** 10.31557/APJCP.2020.21.11.3241

**Published:** 2020-11

**Authors:** Tasuku Okui

**Affiliations:** *Medical Information Center, Kyushu University Hospital, Fukuoka city, Japan. *

**Keywords:** Health status disparities, vital statistics, mortality, cancer, cohort effects

## Abstract

**Background:**

Although socioeconomic statuses affect cancer mortality rates, the specific difference between metropolitan and non-metropolitan areas in Japan has not been evaluated. This study analyzed differences in cancer mortality between metropolitan and non-metropolitan areas in Japan, using an age-period-cohort (APC) analysis.

**Methods:**

Data on cancer mortality from 1999 to 2018 for metropolitan and non-metropolitan areas in Japan were used. Here metropolitan areas were defined as government ordinance-designated municipalities in 1999 and special wards of Tokyo. In addition to general mortality data for all cancer sites, data on mortality for stomach, colorectal, liver, gallbladder, pancreatic, lung, prostate, and breast cancers were used for analysis. A Bayesian APC analysis was administered to the data for each type of cancer for area and for sex-distinguished data. Additionally, the ratios for estimated mortality rate by periods and cohorts between the two areas were calculated.

**Results:**

The age-standardized mortality rate for cancer in all sites in non-metropolitan areas was lower than that in metropolitan areas throughout the analyzed years for both men and women, but the mortality difference decreased during the periods for men. The rates of decrease in mortality rate in cohorts differed for some cancers between the two area types, and the mortality rate ratios of metropolitan compared with non-metropolitan areas decreased for cancer in all sites over the analyzed cohorts for men. Also, the rate of decrease in mortality rate over the cohorts was completely different between the areas for stomach cancer in men and for liver cancer for women.

**Conclusion:**

Mortality rates for cancer in all sites tended to diverge between the two area types in younger cohorts for men, and people in younger cohorts in non-metropolitan areas should take more extensive preventive measures against cancer than their counterparts in metropolitan areas.

## Introduction

Cancer is the foremost cause of mortality in Japan, and according to Ministry of Health, Labour and Welfare of Japan (2020), the mortality rate of the disease increased from 231.6 to 300.7 per 100,000 persons from 1999 to 2018. In particular, the mortality rate for colorectal cancer increased from 28.2 to 40.8 per 100,000 individuals and the mortality rate for lung cancer increased from 41.6 to 59.8 per 100,000 individuals during the same period. Besides, according to the Patient Survey and Vital Statistics in Japan, the estimated number of patients affected by cancer increased from 1010.9 to 1427.2 per 100,000 individuals from 1999 to 2017 (Ministry of Health, Labour and Welfare of Japan, 2020). As the population continues to age, the rate of patients with cancer is expected to increase. The cost burden associated with cancer in Japan is very large, and the national medical expenditure on cancer was 23.76 trillion yen in 2017 (Ministry of Health, Labour and Welfare of Japan, 2017). In addition to direct expenditures on treatment and therapy, the costs associated with cancer includes the mortality or morbidity costs, including loss of income due to going to hospital or decreased labor productivity. Taking into account the costs associated with these factors, the social burden of cancer will become even larger (Haga et al., 2013; Kitazawa et al., 2015; Matsumoto et al., 2015). Therefore, the risk population should be assessed, and appropriate preventive measures should be taken.

Socioeconomic status affects the incidence and mortality of cancer (Williams et al., 1991; Baade et al., 2011; Miranda et al., 2014; Singh and Jamel, 2017), and trends in incidence and mortality vary with socioeconomic factors. Epidemiological studies have investigated the association between socioeconomic status and cancer in Japan (Ueda et al., 2005; Fukuda, Nakamura, et al., 2005), and mortality rates for cancer differ in relation to occupation and municipal economic status (Fukuda, Nakamura, et al., 2005). Disparities in disease mortality between urban and rural areas are often taken as an index of socioeconomic status. In particular, the concentration of population in metropolitan areas, such as government ordinance-designated municipalities, has been a continual feature of Japan for a long period (Ministry of Internal Affairs and Communications, 2020). Although trends in cancer mortality for both metropolitan and non-metropolitan regions have seen change over the years, the difference between these area types has not been subject to recent investigation using government statistics. Additionally, as trends in cancer mortality rates are highly affected by changes in birth cohort effects in Japan (Ito et al., 2011; Okui, 2020), and it is possible that trends in cohort effects differ by area type. 

Age-period-cohort (APC) analysis is widely used to analyze trends in disease mortality (Smith and Wakefield, 2016). An APC analysis is applied to the mortality data over the years, and the mortality and population data for each age group over the years are used for the analysis. The cancer mortality data can be obtained for each age group over the years from the Vital Statistics of Japan, and an APC analysis can be applicable. Using APC analysis, changes in cancer mortality rates by age group over the years can be decomposed into age effects, period effects, and cohort effects. Several APC analyses for mortality rates for various cancer types have already been conducted for Japanese populations using mainly the Vital Statistics in Japan (Takahashi et al., 2001; Ito et al., 2011; Utada et al., 2010; Okui, 2020). However, an APC analysis comparing the mortality rates for metropolitan and non- metropolitan areas has not yet been conducted, and differences in the trends of cohort effects and period effects between the types of areas remain uncertain. Some APC analyses to compare differences in cancer mortality rates between urban and rural areas have been conducted in other countries (Guo and Huang, 2011; Miranda et al., 2014; Sun et al., 2018), and differences in period and cohort effects for specific type of cancer by locality type areas were found. The primary advantage of using APC analysis is that we were able to reveal the cohort effect on the mortality rate. It is known that a decrease in the cancer mortality rate over birth cohorts in the 20th century has led to the decline in the age-standardized cancer mortality rate in Japan (Ito et al., 2011; Okui, 2020). By using APC analysis, we were able to reveal the particular differences in the trend of the cohort effects between the two types of areas.

In this study, we analyzed differences in trends of cancer mortality rate in Japan between metropolitan and non-metropolitan areas using APC analysis.

## Materials and Methods

We used official classifications of government ordinance-designated municipalities and special wards of Tokyo as metropolitan areas in this study. Government ordinance-designated municipalities have more than 500,000 citizens and are allowed more autonomy than other types of municipalities (Ministry of Internal Affairs and Communications, 2020). Although some government ordinance-designated municipalities have been added in recent years, we chose only those that already had this status in 1999 (Sapporo, Sendai, Chiba, Kawasaki, Yokohama, Nagoya, Osaka, Kyoto, Kobe, Hiroshima, Kitakyushu, and Fukuoka) as metropolitan areas because most of the recent increase in the urbanized population has concentrated in these municipalities in particular. Population figures for metropolitan areas and other areas were obtained from the national survey of population, demographics, and households using the basic resident register, including information on sex and age group (Ministry of Internal Affairs and Communications, 2020).

The cancer mortality data from the Vital Statistics in Japan (Ministry of Health, Labour and Welfare of Japan, 2020) from 1999 to 2018 were used for analysis. The Vital Statistics is government statistics in Japan, and all the deaths in Japan are registered to municipalities, and finally put into the Ministry of Health, Labour and Welfare of Japan. In order to assess types of cancer that affected the trend of cancer in all sites, we analyzed the data of representative types of cancer in addition to cancer in all sites. Then, the mortality of cancer in all sites, stomach cancer, colorectal cancer, liver cancer, gallbladder cancer, pancreatic cancer, lung cancer, prostate cancer, and breast cancer were used in the analysis. The International Classification of Diseases (10th Revision) codes for cancer in all sites is C00-97. In addition, the codes for individual cancer types are as follows: stomach, C16; colorectal, C18–20; liver, C22; gallbladder, C23–24; pancreas, C25; lung, C33–34; prostate, C61; and breast, C50 (Ministry of Health, Labour and Welfare of Japan, 2020). Age groups were defined in 5-year units from 40–44 years to 75–79 years in the data for the APC analysis. Also, cohorts were defined for each age group of each year. Then, those who were 75–79 years old in 1999 (i.e., those who were born in 1920–1924) were the first cohort. Through a 1-year shift of the birth years, the age group 40–44 years in 2018 (i.e., those who were born in 1974–1978) was the last cohort.

As the statistical analysis, we calculated the age-standardized mortality per 100,000 persons of each type of cancer for both sexes and types of areas using the direct standardization method. The total population in 1999 was used as the reference population for the calculation of age-standardized mortality. Then, we used a univariate Bayesian Poisson APC model including a heterogeneity term for the analysis (Smith and Wakefield, 2016). For the identifiability of the parameters, the restriction that sum of each age, period, and cohort effect is zero was applied. For the priors of each effect, random walk of the first order was used. Period and cohort effects of each type of cancer were extracted for both types of areas and sexes. Further, the rate ratios for mortality of metropolitan to non-metropolitan areas by period and cohort were calculated for each cancer type and both sexes by using estimated parameters. To estimate the parameters, the Hamiltonian Monte Carlo method was used (Stan Development Team, 2020). All statistical analyses were conducted using R 3.5.1 software (R Core Team, 2020).

## Results

**Figure 1:**
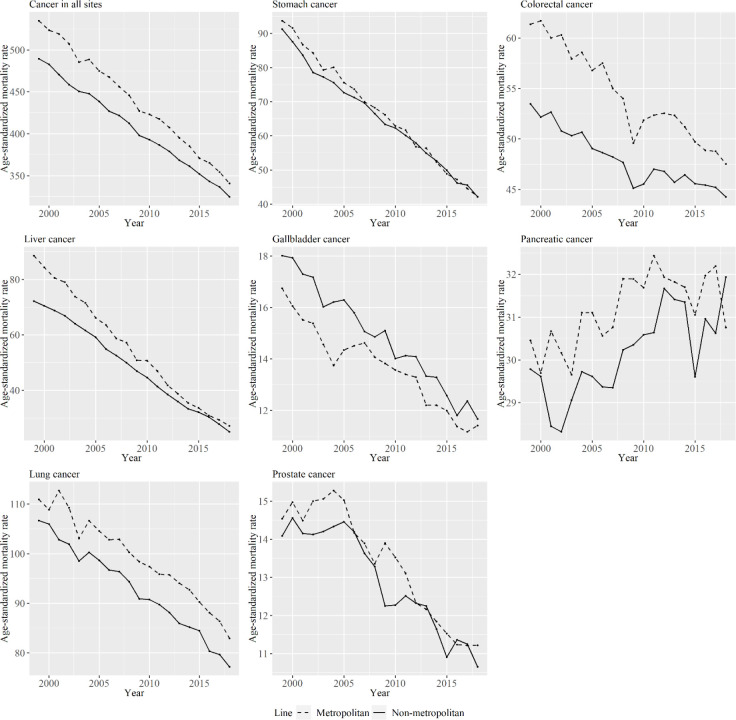
Men’s Age-Standardized Mortality Per 100,000 Persons for Specific Cancer Types from 1999 to 2018 for Metropolitan and Non-Metropolitan Areas. The lines signify the age-standardized mortality per 100,000 persons among periods in metropolitan and non-metropolitan areas. The dashed line represents metropolitan areas, and the solid line represents non-metropolitan areas

Figure 1 shows men’s age-standardized mortality per 100,000 persons for specific types of cancer from 1999 to 2018 for metropolitan and non-metropolitan areas. Most types of cancer, with the exception of pancreatic cancer, decreased over the period of study for both area types. Age-standardized mortality rates for non-metropolitan areas were smaller than those for metropolitan areas throughout the periods in most of cancer types.

**Figure 2:**
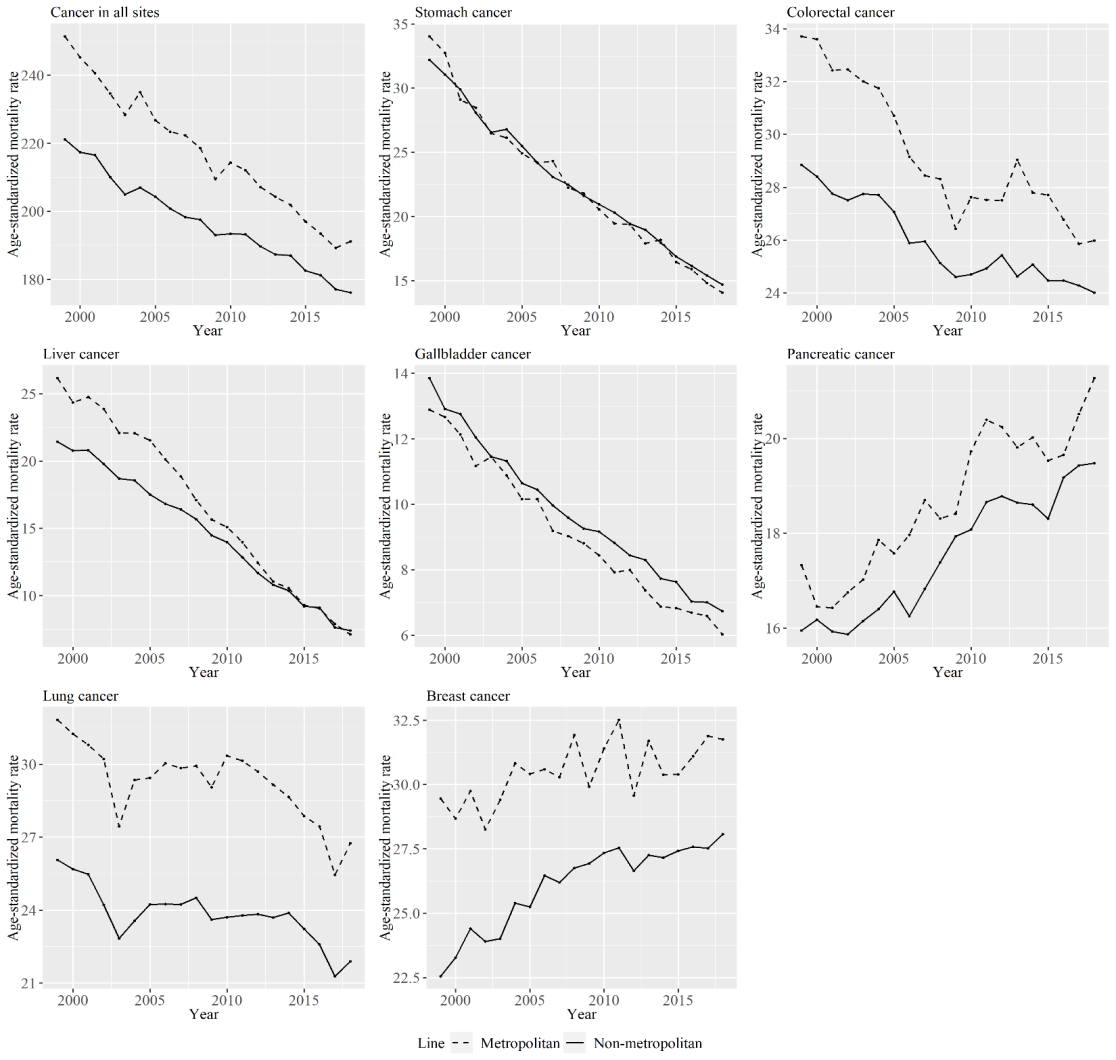
Women’s Age-Standardized Mortality Per 100,000 Persons for Specific Cancer Types from 1999 to 2018 for Metropolitan and Non-Metropolitan Areas. The lines signify the age-standardized mortality per 100,000 persons among periods in metropolitan and non-metropolitan areas. The dashed line represents metropolitan areas, and the solid line represents non-metropolitan areas

Figure 2 shows women’s age-standardized mortality per 100,000 persons for specific types of cancer from 1999 to 2018 for metropolitan and non-metropolitan areas. Mortality for many cancer types decreased over the period of study for both areas area types, although that for pancreatic and breast cancer did not. The difference in mortality for cancer in colorectal cancer and liver cancer between metropolitan and non-metropolitan areas decreased from 1999 to 2018. Additionally, the increase in breast cancer mortality was larger for non-metropolitan areas than for metropolitan areas.

**Figure 3:**
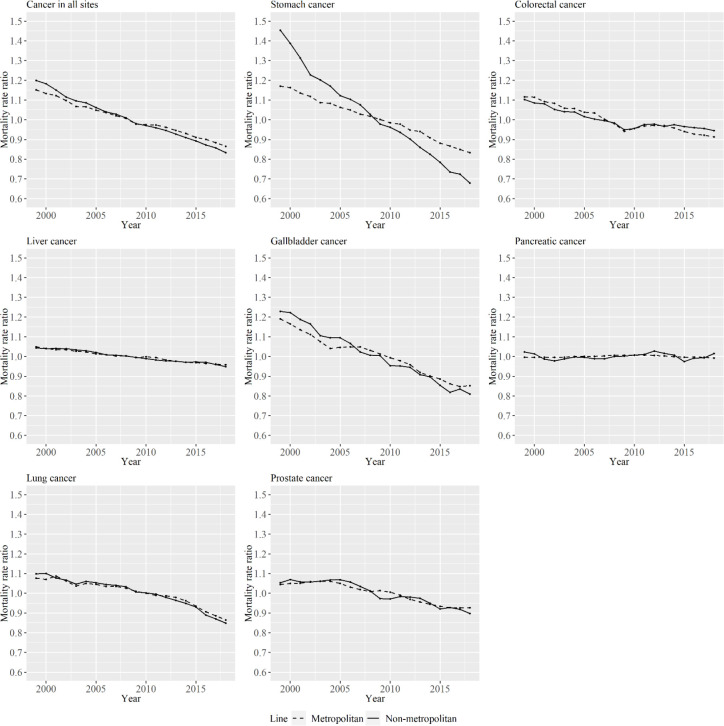
Period Effects for Metropolitan and Non-Metropolitan Areas for Specific Cancer Types among Men. The lines signify the point estimates for mortality rate ratio among periods in metropolitan and non-metropolitan areas. The dashed line represents metropolitan areas, and the solid line represents non-metropolitan areas

Figure 3 shows the period effects of APC analysis for metropolitan and non-metropolitan areas according to cancer type among men. There was a lager decrease in period effects over the period of study in non-metropolitan areas than in metropolitan areas for stomach cancer.

**Figure 4:**
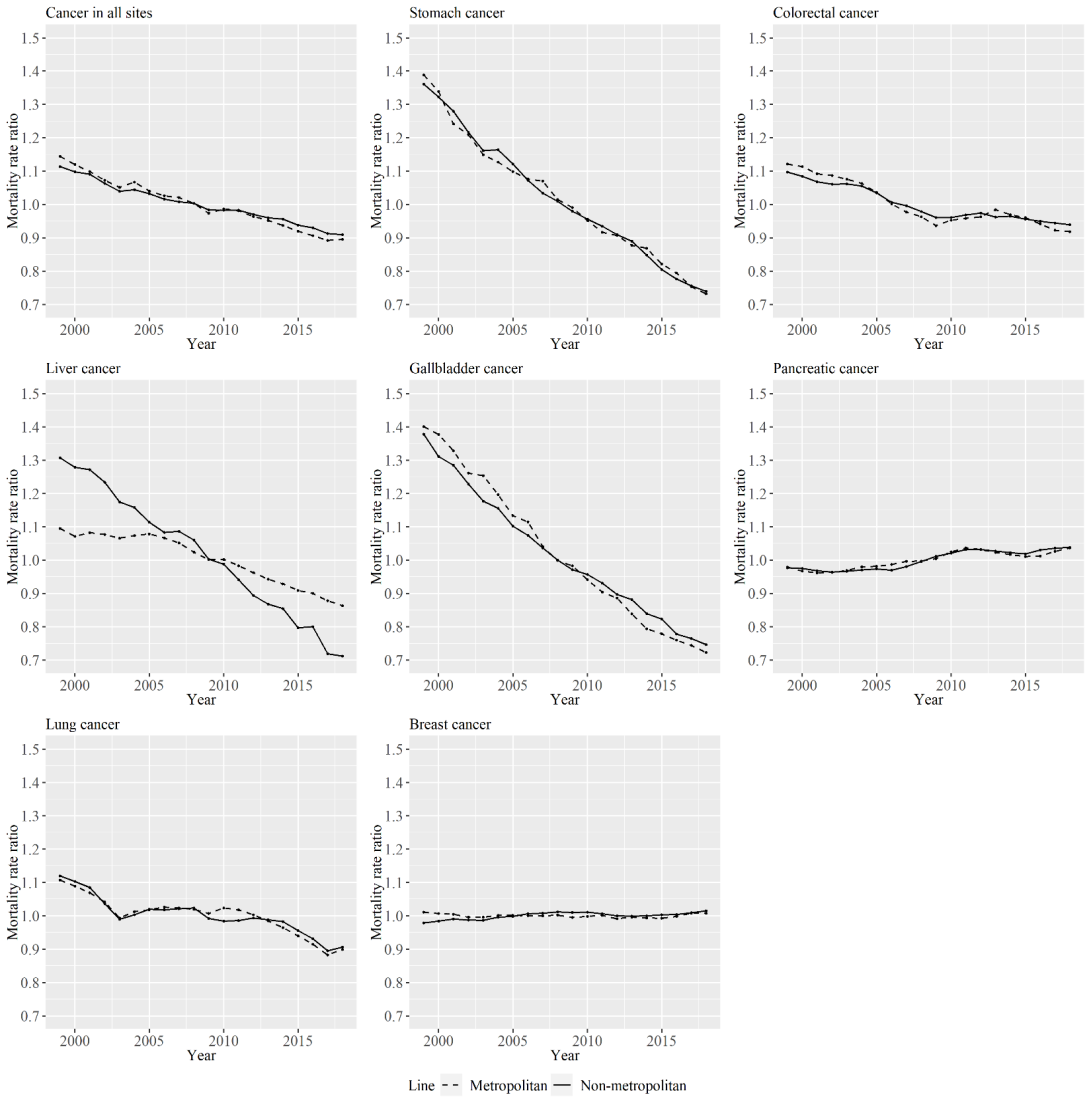
Period Effects for Metropolitan and Non-Metropolitan Areas for Specific Cancer Types among Women. The lines signify the point estimates for mortality rate ratio among periods in metropolitan and non-metropolitan areas. The dashed line represents metropolitan areas, and the solid line represents non-metropolitan areas

Figure 4 shows the period effects in APC analysis for metropolitan and non-metropolitan areas among women by cancer type. The decrease in period effects during the period was larger in non-metropolitan areas than in metropolitan areas for liver cancer.

**Figure 5:**
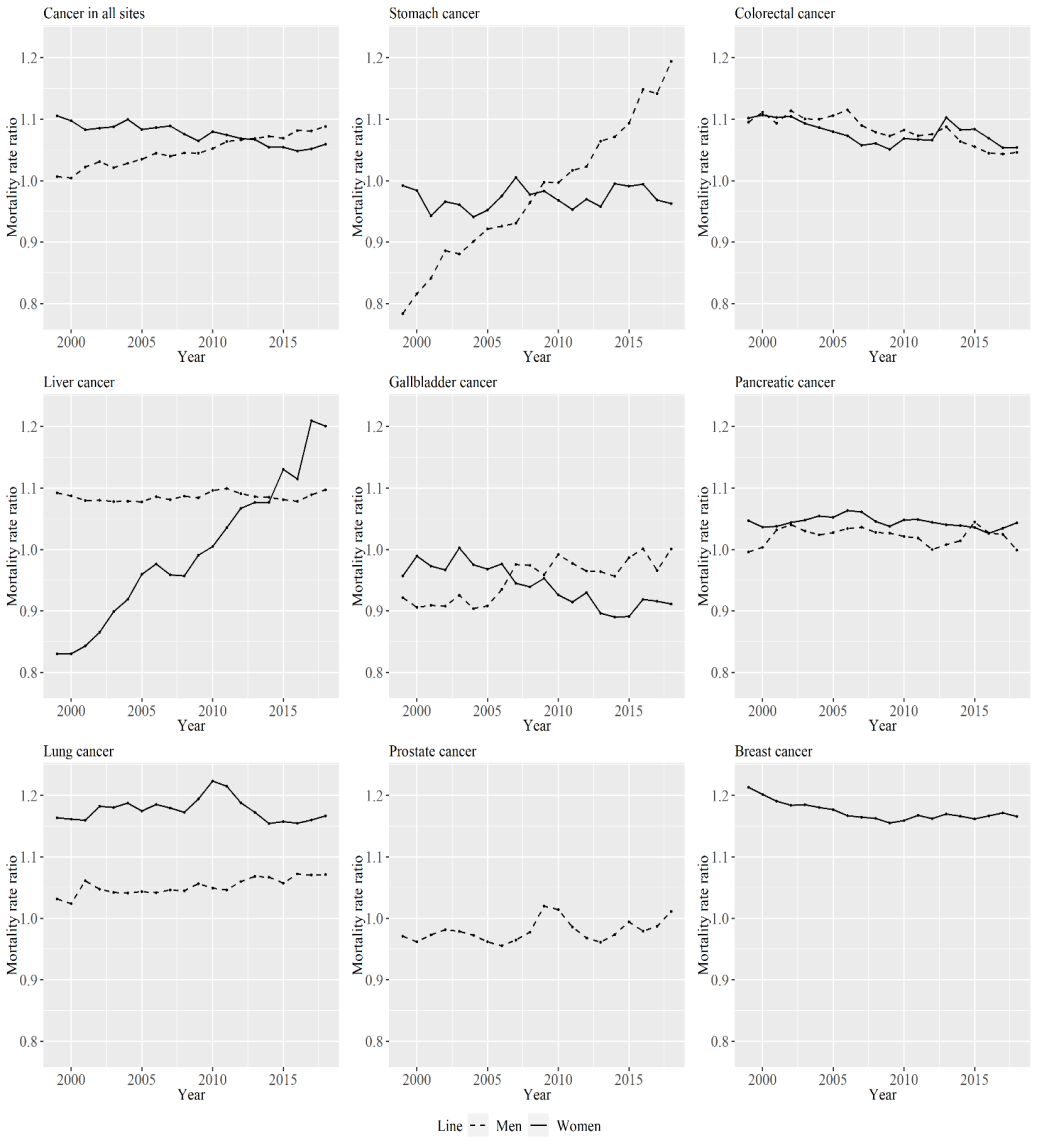
Mortality Rate Ratios of Metropolitan Areas Compared with Non-Metropolitan Areas for Specific Cancer Types by Period for Men and Women. The lines signify the point estimates for the mortality rate ratios in men and women. The dashed line represents men, and the solid line represents women

Figure 5 shows the mortality rate ratios of metropolitan areas compared with non-metropolitan areas for the specific types of cancer in each period for men and women. The mortality rate ratios varied in relation to cancer type and sex. The mortality rate ratio for cancer in all sites between metropolitan and non-metropolitan areas was above one in both men and women throughout the analyzed periods.

**Figure 6:**
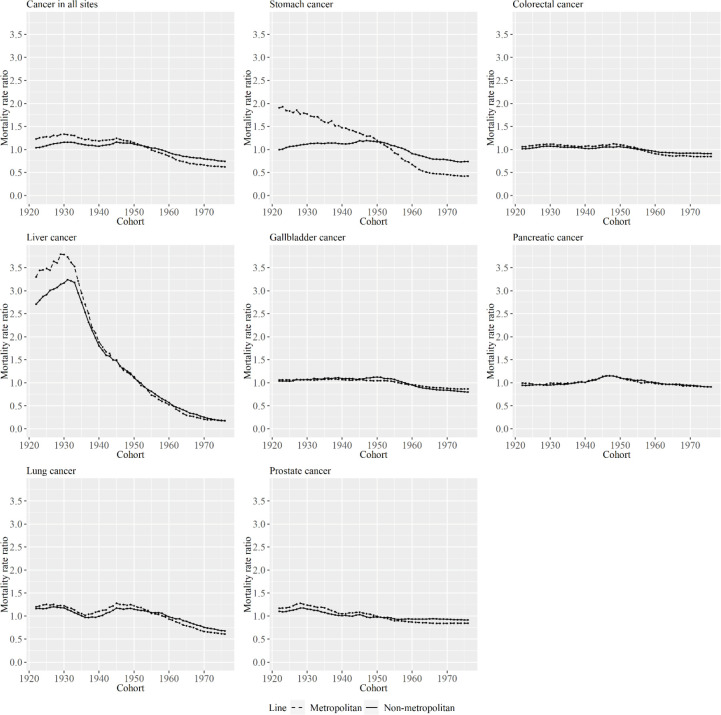
Cohort Effects for Metropolitan and Non-Metropolitan Areas for Specific Cancer Types among Men. The lines signify the point estimates for mortality rate ratio among cohorts in metropolitan and non-metropolitan areas. The dashed line represents metropolitan areas, and the solid line represents non-metropolitan areas

**Figure 7:**
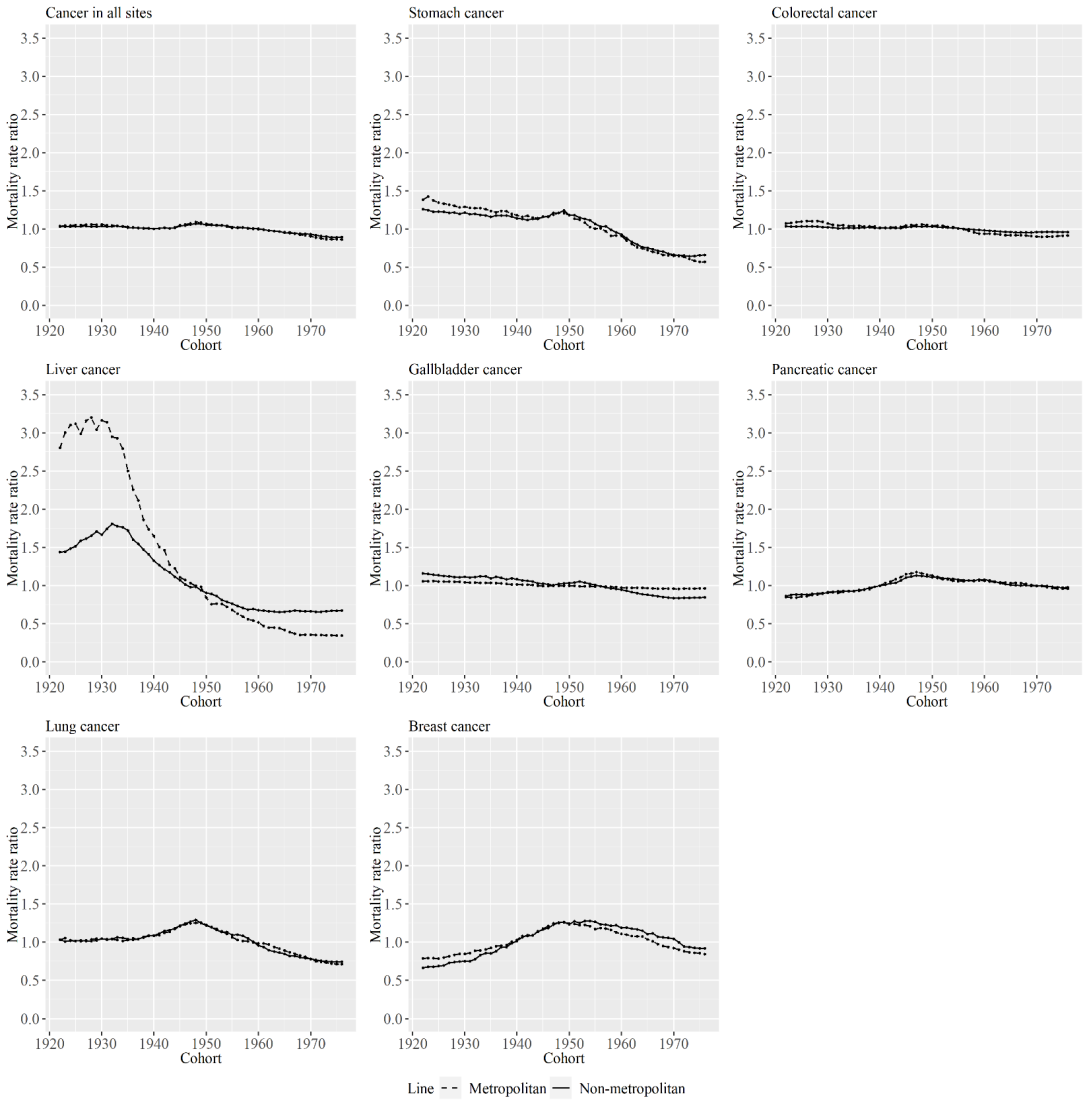
Cohort Effects for Metropolitan and Non-Metropolitan Areas for Specific Cancer Types among Women. The lines signify the point estimates for mortality rate ratio among cohorts in metropolitan and non-metropolitan areas. The dashed line represents metropolitan areas, and the solid line represents non-metropolitan areas

Figures 6 and 7 show cohort effects of APC analysis for metropolitan and non-metropolitan areas, in relation to specific cancer types among men and women. The decrease in cohort effects was larger in metropolitan areas than in non-metropolitan areas for most cancer types, except for pancreatic cancer and gallbladder cancer in men and women. In particular, the decrease in cohort effects was entirely different between area types for stomach cancer in men and for liver cancer for women.

**Figure 8:**
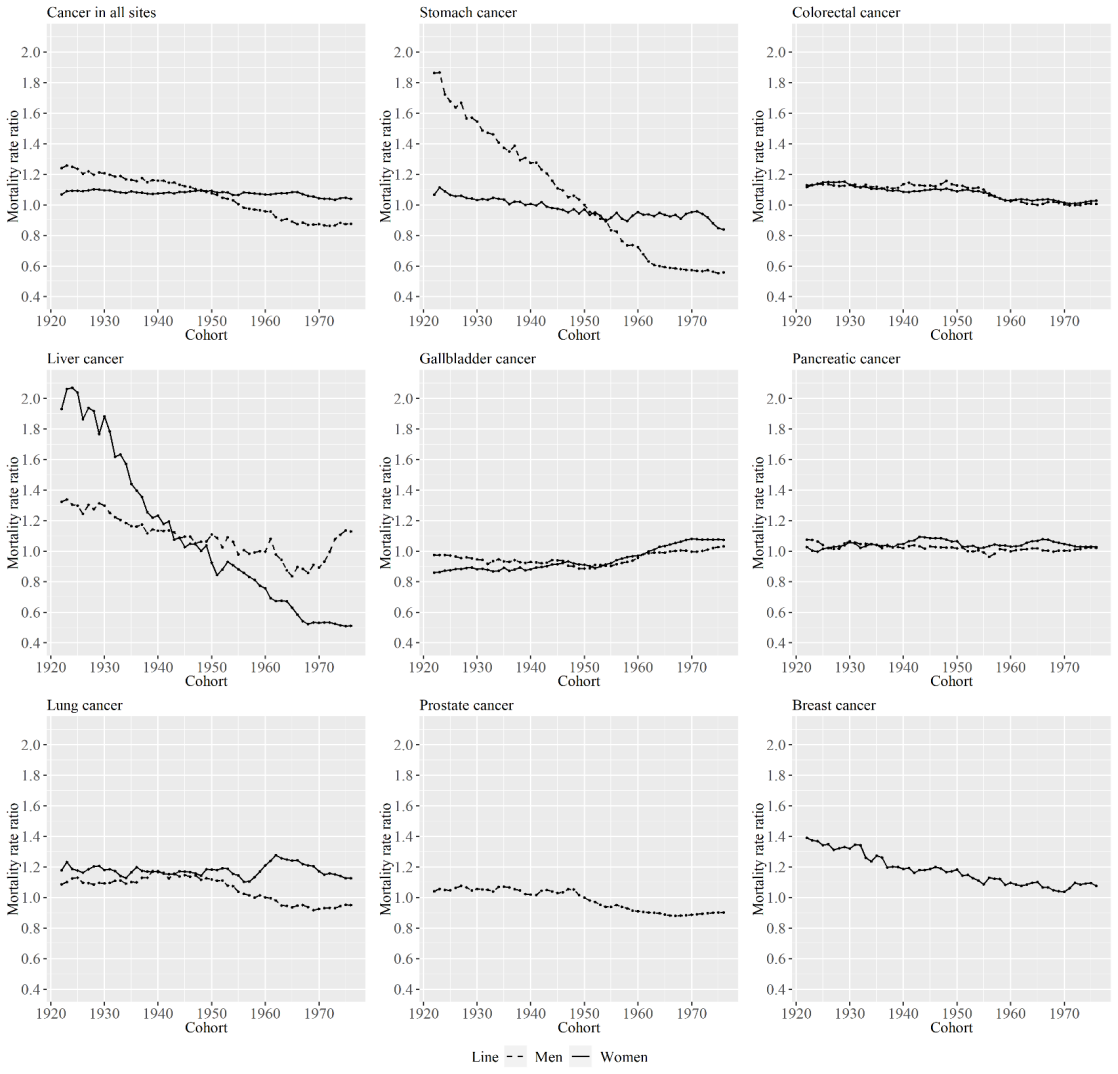
Mortality Rate Ratios of Metropolitan Areas Compared with Non-Metropolitan Areas for Specific Cancer Types by Cohort for Men and Women. The lines signify the point estimates for the mortality rate ratios in men and women. The dashed line represents men, and the solid line represents women

Figure 8 shows mortality rate ratios of metropolitan areas compared with non-metropolitan areas by type of cancer areas in each cohort by sex. Decreases in the ratio were particularly evident in men for cancer in all sites and stomach cancer, and in women, the ratios for liver and breast cancer particularly decreased across cohorts. Although the ratio for cancer in all sites was above one in cohorts born in early 1920s for men, it fell below 1 for more recently born cohorts. The trends in the mortality rate ratio for cancer in all sites in men and women were linked with the mortality rate ratio of stomach cancer.

## Discussion

From the results shown in Figures 1 and 2, the relative ranking of age-standardized mortality for some cancer types between metropolitan and non-metropolitan areas varied by period. However, age-standardized mortality for cancer in all sites was smaller in non-metropolitan areas than in metropolitan areas throughout the analyzed periods. In a previous study that investigated the association between socioeconomic position and cancer mortality in Japanese municipalities in 1993–1998, standardized mortality rates for cancer in all sites in municipalities with lower socioeconomic status was lower than in high socioeconomic status (Fukuda, Nakamura, et al., 2005). That result is consistent with the findings of this study. The cause of this phenomenon is said to be that most cancers are caused by risky behaviors, and these behaviors are more likely to be observed among people living in urban areas (Gersten et al., 2002; Fukuda, Nakamura, et al., 2005). In the same study by Fukuda et al., clusters of breast cancer and colon cancer were observed mostly in urban-rich areas (Fukuda, Umezaki, et al., 2005). This study also show that age-standardized mortality rates of colorectal and breast cancer were larger in metropolitan areas. However, as seen in Figures 1 and 2, the decreasing rate in age-standardized mortality rate for cancer in all sites was larger in metropolitan than in non-metropolitan areas in the analyzed periods for men. The decreasing rate in age-standardized liver and colorectal cancer mortality was particularly different between the area types in men and women, and the increase in breast cancer was particularly different between area types. Here, we focus on stomach, liver, and breast cancer because the trends of cohort effects of these types were particularly different between the two area types, as shown in the figures.

In relation to stomach cancer, although the decreasing rate in the period effects was larger in non-metropolitan areas for men, the decreasing rate in the cohort effects were larger in metropolitan areas for men and women, and the estimated mortality rate for more recently born cohorts in metropolitan areas was smaller than for that in non-metropolitan areas for men and women. The age-standardized incidence rate for stomach cancer decreased beginning in births in 1975 (Qiu et al., 2009). The decreased incidence and mortality rate for stomach cancer in Japan is usually explained through the decreased prevalence of Helicobacter pylori and salt intake (Tanaka et al., 2012; Wang C et al., 2015), and the prevalence of Helicobacter pylori is related to trends in cohort effects. The prevalence of Helicobacter pylori has been shown to have decreased in more recently born cohorts, particularly those born after 1950s (Ueda et al., 2014). The acquisition of Helicobacter pylori infection occurs mostly under the age of 5 years, and infection status is closely tied to the sanitary environment (Ueda et al., 2014). Economic growth has been shown to be associated with a decrease in the prevalence also in China (Yu et al., 2017). Therefore, the change in sanitary conditions is different between the two area types. When we adjusted for cohort effects, period effects became larger in non-metropolitan areas for men. This means that the rate of decrease in mortality for each age group or cohort over the years was higher in non-metropolitan areas. Similarly, the rate of decrease in mortality across cohorts was higher in metropolitan areas. Although the reason for the higher decrease in mortality rate for non-metropolitan areas for men is unknown, the difference in the screening rate might have affected the results. This assertion is plausible since it is a known fact that screening rate is associated with stomach cancer mortality rate in Japan (Yoshida et al., 2010).

As for liver cancer, although period effects decreased, particularly in non-metropolitan areas for women, cohort effects particularly decreased in metropolitan areas for women. The reduction in age-standardized liver cancer mortality rates can be attributed to the decreased prevalence of hepatitis viruses (Tsukuma et al., 2005). The age-standardized incidence rate of liver cancer in Japan decreased beginning in the late 1990s (Katanoda et al., 2015). The largest cause of liver cancer in Japan is the Hepatic C virus (HCV) (Tsukuma et al., 2005), and the decrease in the incidence rate in Japan is due to a decreased incidence rate of the cancer in the 50–69 age group among women due to the decline of HCV infection (Qiu et al., 2009). The decrease in incidence rate of liver cancer in the 50–69 age group among women is particularly evident in non-metropolitan areas. It is important to investigate whether incidence trends for liver cancer will continue to differ between the area types in the future. It has also been shown that the prevalence of HCV antibodies is highest in birth cohorts born in around 1935, and the prevalence decreases in younger generations (Tsukuma et al., 2005). The decrease in prevalence of the HCV is likely be the result of decreased blood infection of contaminated blood (Gersten et al., 2002). The difference in the decrease in the cohort effect for liver cancer between the area types was observed in women, and blood transfusion due to the childbirth may be related to this.

For breast cancer, the estimated mortality rate in metropolitan areas was higher than in non-metropolitan areas in al the periods and cohorts. Breast cancer prevalence tends to be higher in urban areas generally (Mead, 2008), and the result in this study is consistent with this tendency. However, the ratio between area types decreased over birth cohorts, as shown in Figure 8. Cohort effects for mortality decreased for cohorts born in approximately 1950. It has been shown that the cohort effect for the incidence rate of breast cancer also decreased across time points in one prefecture of Japan (Ito et al., 2011). The risk factors related to the cohort effects for breast cancer mortality include lifestyle (greater use of alcohol and/or cigarettes) and dietary habits (greater consumption of a Western-style diet, including high intake of dietary fat and/or calories) (Wang Z et al., 2015). Trends in cohort effects for pancreatic cancer were relatively similar to those for breast cancer, as shown in Figure 7. The possible risk factors for pancreatic cancer also include smoking and obesity (Nakamura et al., 2011; Ilic et al., 2016), so trends in some of these factors may be different between the area types across cohorts. In contrast, cohort effect on obesity prevalence for women was shown to increase for cohorts born between 1965 and 1974 (Yamakita et al., 2015), other factors than obesity prevalence is considered to be related to the result. The increasing awareness of breast cancer is another possible explanation for this trend (Wang et al., 2015). Additionally, early access to medical care and health insurance positively contributed to survival of patients with breast cancer (Wang et al., 2015). Furthermore, it is noteworthy that the decline in the cohort effect was also observed in European countries. This decline is attributed to the following factors: introduction of effective therapies, progress in surgery and radiotherapy, and mammography screening (Rosso et al., 2018). Hence, a disparity in the access to medical resources or early detection of breast cancer (and other types of cancer) plays a major role in the mortality rates in these areas.

This study had some limitations. First, APC analysis is a descriptive method, meaning that the precise reason for any given difference between the area types must be acknowledged to be uncertain. Second, although metropolitan areas were defined as those that were government ordinance-designated municipalities in 1999 and the special wards of Tokyo, differences can be seen in cancer mortality rates even among the metropolitan areas. By analyzing data for each of the metropolitan area, future work may be able to precisely identify differences among these areas. However, by using APC analysis, we were able to reveal the differences in the trend of the cohort effects between the two types of areas. Although only the trends of age-standardized cancer mortality rates are often exploited for describing the mortality trend (Qiu et al., 2009), the mortality rates vary largely depending on birth cohorts in particular for stomach and liver cancer, as shown in the current study. Therefore, it would be meaningful to also analyze the trend of cohort effect for analyzing the regional differences in the cancer mortality rates in the future.

Finally, the decrease in age-standardized mortality rates for cancer in all sites was larger in metropolitan areas than non-metropolitan areas from 1999 to 2018 for men. Although age-standardized cancer mortality in Japan was known to be higher in urban areas (Fukuda, Nakamura, et al., 2005), this tendency has been changing in recent years for men. In particular, the ratio of mortality between metropolitan and non-metropolitan areas decreased across cohorts for cancer in all sites for men. If this tendency continues, the relationship between the area types may be inverted. Thus, people in younger cohorts in non-metropolitan areas require greater preventive measures against cancer than their counterparts in metropolitan areas for men.
